# The Role of ND10 Nuclear Bodies in Herpesvirus Infection: A Frenemy for the Virus?

**DOI:** 10.3390/v13020239

**Published:** 2021-02-03

**Authors:** Behdokht Jan Fada, Eleazar Reward, Haidong Gu

**Affiliations:** Department of Biological Sciences, Wayne State University, Detroit, MI 48202, USA; ga2861@wayne.edu (B.J.F.); reward.eleazar@wayne.edu (E.R.)

**Keywords:** herpesvirus, ND10, PML nuclear bodies, virus-host interaction, antiviral defense

## Abstract

Nuclear domains 10 (ND10), a.k.a. promyelocytic leukemia nuclear bodies (PML-NBs), are membraneless subnuclear domains that are highly dynamic in their protein composition in response to cellular cues. They are known to be involved in many key cellular processes including DNA damage response, transcription regulation, apoptosis, oncogenesis, and antiviral defenses. The diversity and dynamics of ND10 residents enable them to play seemingly opposite roles under different physiological conditions. Although the molecular mechanisms are not completely clear, the pro- and anti-cancer effects of ND10 have been well established in tumorigenesis. However, in herpesvirus research, until the recently emerged evidence of pro-viral contributions, ND10 nuclear bodies have been generally recognized as part of the intrinsic antiviral defenses that converge to the incoming viral DNA to inhibit the viral gene expression. In this review, we evaluate the newly discovered pro-infection influences of ND10 in various human herpesviruses and analyze their molecular foundation along with the traditional antiviral functions of ND10. We hope to shed light on the explicit role of ND10 in both the lytic and latent cycles of herpesvirus infection, which is imperative to the delineation of herpes pathogenesis and the development of prophylactic/therapeutic treatments for herpetic diseases.

## 1. Introduction

Nuclear domains 10 (ND10) are dynamic macromolecular structures that assemble in the nucleoplasm of many types of mammalian cells [1,2]. They were first identified as unique nuclear dots recognized by autoimmune antibodies that were distinguishable from other known nuclear bodies [3]. These discrete spherical dots were designated as ND10 based on their average number of 10 loci per nucleus in various cultured cells [3,4]. More interestingly, the size and number of ND10 were found to increase with interferon (IFN) treatment and diminish upon herpes simplex virus (HSV) infection [4,5]. These discoveries immediately linked this new type of nuclear body to host antiviral response and raised enormous interest in the field of virology [1,6,7,8].

In that same era, parallel studies investigating the acute promyelocytic leukemia (APL) etiology revealed that a chromosome t(15;17) translocation that led to a fusion of promyelocytic leukemia (PML) protein to the retinoic acid receptor α (RARα) was the direct cause of APL [9,10]. Studies on PML quickly identified PML nuclear bodies (PML-NBs), in which the wild-type PML was a constant constituent. Further experiments showed that the introduction of the PML/RARα fusion protein to normal cells disrupted PML-NBs, whereas treatment with the pharmacological doses of retinoic acid (RA) restored PML-NBs in the APL-derived NB4 cells [11,12]. Since the clinical RA administration was known to induce blast differentiation and complete remission in APL patients, PML was thereby classified as a tumor suppressor, and oncogenic regulation has since been considered an important function of PML-NBs [13,14,15,16].

With the extensive interest invested in characterizing the composition and functions of these novel nuclear bodies, people quickly realized that ND10 bodies and PML-NBs represented the same set of macromolecular structures, for which we follow the herpes virologists’ tradition and call them ND10 from this point on. Besides their implication in antiviral defense and tumor suppression, the ND10 structures are known to function in many cellular pathways, including transcription/translation regulation, DNA damage response, cell cycle regulation, and apoptosis and senescence regulation [1,17,18,19]. So far, more than 150 component proteins are known to associate with ND10 bodies [20]. Among them, some proteins are permanent residents such as PML, whereas many others are transiently associated with ND10 in response to various cellular cues [20,21]. The majority of ND10 components are regulatory proteins with multiple functions in key cellular pathways. The diversity and importance of ND10 proteins in a cell’s life have kept these nuclear bodies in the scientific spotlight for decades, yet how ND10 bodies dynamically shift the components to orchestrate a specific function is still not well understood. Recent studies on both the virology and cancer biology fronts have implied that ND10 may play dual roles and serve as a frenemy in establishing the viral replication and tumor malignancy [22,23], which have attracted further interest in the complex functions of ND10.

Theoretically, viruses depend on the host cell machineries to complete their life cycles, so they must have developed delicate means to differentiate friends from foes in the host. ND10 nuclear bodies, with the diverse array of positive and negative gene regulators enriched in the loci, are known to mount defenses to downregulate viral expression, but they also contain abundant gene activators waiting to be harnessed by the virus. The aggregation of both beneficial and inimical factors within ND10 provides us a unique opportunity to understand the intimate frenemy relation of a host to the virus, which is the molecular foundation for viral infection and pathogenesis. In this review, we evaluate the recent discoveries of pro-viral phenomena of ND10 components in human herpesvirus infection, along with their traditional antiviral mechanisms. We hope this review will shine light on the current understanding of the molecular bases of virus-host interactions.

## 2. ND10 Composition and Biogenesis

As mentioned earlier, the ever-growing list of functionally diverse proteins associated with ND10 has implied promiscuous functions for these nuclear bodies. The constant ND10 constituents are likely the leading factors that organize the various functions. Here we briefly introduce the few well-known ND10 constituents most relevant to many virology studies discussed in this review.

PML, also known as TRIM19, is a member of the tripartite motif (TRIM)-containing protein family that serves as the ND10 organizer. The conserved TRIM located in the N-terminus of PML, also known as the RING-BOX-Coiled Coil (RBCC) domain [24], mediates the PML oligomerization and is the foundation for ND10 formation [25]. PML has at least seven isoforms generated by alternative splicing of a single gene. All PML isoforms share the RBCC containing N-terminus but vary in their unique C-termini [24]. While all PML isoforms can be induced by an IFN treatment [26], the expression profile of these isoforms is not uniform, with PML I and II at levels higher than the others [27]. All isoforms except PML VII contain a conserved nuclear localization signal (NLS) and work as nuclear proteins. PML is post-translationally modified by small ubiquitin-like modifier (SUMO) on several lysine residues in a cell-cycle-dependent manner [28]. It also has a SUMO interaction motif (SIM) that allows the protein to interact with SUMOylated partners. The SUMO–SIM interactions among ND10 components are the main driving forces that mediate the protein networking within ND10 [2,29]. Since the discovery of PML as a tumor suppressor, many important cellular functions have been associated with this protein. Among them, PML is known to be involved in cell cycle regulation, senescence, apoptosis, regulation of angiogenesis, regulation of mRNA translation, chromatin remodeling, and antiviral defenses [21,30,31,32]. Lately, increasing evidence has shown differential functions for the individual PML isoforms. For example, in the gene locus of major histocompatibility class (MHC) I, PML interacts with SATB1 (specific AT-rich binding protein 1), a protein involved in the organization of higher-order chromatin architecture through loop formation. Knockdown of individual PML isoforms differentially changes the chromatin loop formation and subsequently leads to activation, suppression, or no change in the expression of genes located in the MHC I locus [33] (for details on the differential functions of PML isoforms, see Reference [34]).

Members of the speckled protein (SP) family are another group of dominant residents in the ND10 bodies [35,36], including Sp100, Sp110, and Sp140, all of which have different isoforms resulting from alternative splicing [37]. Similar to PML, SPs are IFN-stimulated genes (ISGs), function in the nucleus, and can be modified by SUMOylation for their recruitment to ND10 [5,38,39,40]. Additional functional domains of SPs include a SAND domain that binds to DNA, a plant homeodomain (PHD) that recognizes histone methylation, and a bromodomain (BRD) that reacts to histone acetylation [37]. Because of their ability to interact with DNA and the modified histones, SPs are known as chromatin readers in gene transcriptional regulation and are believed to participate mainly in immune response. For example, patients with primary biliary cholangitis have autoimmune antibodies against Sp100 in their sera [41], whereas loss-of-function mutations of Sp110 are linked to immunodeficiency [42]. SPs likely regulate the immune response through chromatin remodeling, but the exact mechanisms are still elusive. Similar to PML isoforms, SP isoforms also have differential functions in immune regulation, inasmuch as Sp110 B and C are found to suppress the NF-κB responsive promoters, while Sp110 A can enhance them [43].

Another classical component of ND10 is the death domain associated protein (Daxx) [44]. Daxx has two SIM domains located in the N- and C-termini that mediate its interaction with SUMOylated proteins such as PML [45,46]. Besides the full-length Daxx, two C-terminal alternatively spliced isoforms, Daxx-β and Daxx-γ, exist that are less localized to the ND10 bodies due to the loss of the C-terminal SIM [47,48]. Daxx is a well-known H3.3 histone chaperone that interacts with the histones H3.3/H4 dimer through its histone binding domain (HBD) [49,50]. In its central region, Daxx has a four-helix bundle (4HB) that allows the protein to interact with many partners such as transcription factors, chromatin remodelers, and histone modifiers [51]. One of these partners is the chromatin remodeler alpha-thalassemia/mental retardation X-linked syndrome protein (ATRX) [52]. The Daxx/ATRX complex can deposit H3.3 into the heterochromatin regions and participate in the regulation of apoptosis and DNA damage response [51,53]. Dysregulation of Daxx/ATRX has been observed in many types of cancer (for a detailed review, see Reference [51]).

PML plays an essential structural role in the formation of ND10 and is therefore regarded as the ND10 organizer or ND10 scaffold protein [2]. In mouse PML-/- embryonic fibroblasts, Sp100 is found dispersed throughout the nucleus, while Daxx aggregates at condensed chromatin that is morphologically different from the ND10 structures [45]. However, in human PML-/- epithelial cells, Xu et al. found that Sp100 and Daxx colocalized with each other but in condensates that did not resemble ND10 in either size or number [22], suggesting that the ND10 formation and ND10 dynamics vary in different species or types of cells. Further analyses have shown that for ND10 to take shape, PML molecules first form an outer shell via the self-oligomerization mediated by the RBCC domain [25,40] and then strengthen the PML–PML intermolecular interactions through SUMO–SIM interactions [54]. The secondary enhancement of SUMO–SIM interactions between the PML proteins is dispensable in the nucleation of ND10 formation because a PML mutant lacking the SUMO/SIM sites is still able to form the spherical shell of ND10 [40]. However, SUMO–SIM interactions are required for the recruitment of other components to the inner core of ND10 [40,55]. The recent discovery of multivalent SUMO–SIM interactions as a driving force for the formation of membraneless condensates via liquid-liquid phase separation (LLPS) [56,57] has triggered a hypothesis that ND10 bodies most likely form through LLPS [13,56,58]. Since the SUMO–SIM interactions are more important for the inner core of ND10 rather than the outer shell, the biogenesis of ND10 may be more complicated than can be explained by LLPS. Interestingly, many viral proteins such as ICP0 of HSV-1 [59], IE1 of HCMV [60], BZLF1 of EBV [61], and Rta of KHSV [62], all of which target ND10 to counteract the suppression on viral genome, are SUMOylated or contain SIMs and rely on the SUMO–SIM interactions to be recruited to ND10.

## 3. Brief Overview of the Dual Role of ND10 in Tumorigenesis

The lack of functional PML and the abnormality of ND10 have been found not only in hematologic cancers but also in the various solid tumors including breast, colorectal, prostate, lung, and bladder cancers [63,64,65], suggesting the critical role of ND10 in tumor suppression. Much of the tumor suppression activity of ND10 is mediated through the numerous permanent and transient components of ND10 that regulate the cell cycle, DNA damage response, apoptosis, and senescence [1,17,18,19]. For example, the aforementioned Daxx protein promotes apoptosis and suppresses cell proliferation by repressing the expression of anti-apoptotic genes including Bcl2 and survivin through enhancing the heterochromatin marks on their promoters [51,66]. Furthermore, the first-ever identified tumor suppressor, p53, and the p53 modifiers such as the p53 activating kinase HIPK2, the p53 E3 ubiquitin ligase MDM2, and the p53 deacetylase SIRT1 are transient components recruited to ND10 upon specific inductions [67,68,69], suggesting that the critical role of p53 in apoptosis and cell cycle checkpoint is regulated through cross-talking with ND10. For example, upon DNA damage, ND10 nuclear bodies sequestrate MDM2 and prevent the MDM2-mediated degradation of p53. Consequently, the upregulation of p53 mediated by ND10 promotes apoptosis and inhibits cell proliferation [69,70]. Additional ND10 components associated with the heterochromatin formation and cell senescence regulation, such as retinoblastoma protein (pRb) and HIRA, are also known to inhibit cell proliferation by inducing senescence and further underpin the tumor suppression role of the ND10 nuclear bodies [71,72].

Although the complete or partial loss of functional PML is a hallmark for various tumors [63,65], higher expression of PML has also been reported in tumors such as ovarian carcinoma and triple-negative breast cancer (TNBC) [23,73]. Interestingly, recent results showed that PML knockdown in cultured cells derived from these tumors induced apoptosis in the ovarian carcinoma cells and senescence response in the TNBC cells and thereby inhibited the cell proliferation in tissue culture or mouse xenograft [23,73], suggesting the pro-tumor roles of PML and ND10 in these particular cancers. Further investigation in the TNBC cells demonstrated that arsenic treatment, which triggers PML degradation [74], did not elicit the senescence response like that of PML knockdown [23], indicating that distinctive methods of removing PML can lead to differential cellular response, something analogous to what has been shown in the HSV-1 studies, which are discussed in the next section. More complex pro-cancer functions of PML have been found in the maintenance of leukemia stem cells, neuroblast migration, and TNBC metastasis [75,76,77]. Seemingly, the diverse ND10 components may regulate the different aspects of tumorigenesis in a cancer-type-dependent manner.

As in cancer research, ND10 nuclear bodies are traditionally considered as antiviral restrictions, in which the main components such as PML, Sp100, and the Daxx/ATRX complex are involved in inhibiting viral DNA transcription/replication via IFN response or chromatin repression. However, the fact that viral gene expression starts with the viral DNA located at ND10 [78] has long raised the suspicion that ND10 nuclear bodies may also provide functions favorable to viral replication. Here we summarize recent literature about ND10 effects in human herpesviruses to delineate the dual functions of ND10 in the virus-host interactions.

## 4. Dual Role of ND10 in α-Herpesvirus Infection

### 4.1. HSV Lytic Infection

Herpes simplex viruses 1 and 2 are members of the Alphaherpesvirinae subfamily that establish lytic infection in epithelial cells and then the lifelong latency in neuronal cells. Although the prevalence and latency sites show some differences between HSV-1 and HSV-2, many of the molecular events found in HSV-1 can be recapitulated in HSV-2. As mentioned above, the antiviral functions of ND10 are first implied by the disappearance of ND10 in HSV-1 infection [4]. Most of the mechanistic studies of ND10 functions also come from understanding the relations between HSV-1 and ND10. First, HSV-1 DNA, upon entering the nucleus, is immediately found in the vicinity of ND10 loci in the high multiplicity of infection (MOI) [79]. However, results at low MOI showed that in newly infected cells at the edge of a developing plaque, components of ND10 relocated to the viral DNA entry site near the nuclear periphery [80,81]. The convergence of ND10 proteins towards the incoming DNA upon its entry reflects the host effort to immediately dampen the expression of invading DNA as part of the cell’s intrinsic antiviral defenses. Interestingly, nascent ICP0 (infected cell protein 0), an immediate-early (IE) protein of HSV-1, also colocalizes with ND10 upon its synthesis [82]. ICP0 has an E3 ubiquitin ligase activity, which recognizes PML and Sp100 and targets them for proteasomal degradation [83,84,85]. The loss of the ND10 organizer, PML, leads to the disruption of ND10 structures and the dispersal of ND10 components [84,85,86]. The ICP0-mediated ND10 destruction is a critical step in early HSV-1 infection, inasmuch as the replication of an ICP0-null virus or a recombinant virus with ICP0 incapable of ND10 colocalization is largely defective in viral growth at low MOI [87,88,89]. Moreover, Everett et al. showed that depletion of PML, Sp100, Daxx, or ATRX, individually or in combination, improved the growth of the ICP0-null virus [90,91,92,93], indicating that ND10 components are repressive to viral transcription/replication and ICP0 is a major viral counteraction that alleviates the ND10 restrictions.

Although ND10 nuclear bodies are viewed as a part of the cellular antiviral defense systems, a few recent studies have demonstrated a more complex side of ND10. For example, an independent PML knockdown assay conducted by Knipe and colleagues showed a decrease of viral yield for an ICP0-null virus derived from a different HSV-1 strain [94], contradictory to the results of Everett et al. as described above [90]. The contradiction may be the reflection of a strain-dependency in the ICP0–ND10 interaction, or more likely, experimental variations in the two different knockdown methods used in those reports, where the Everett group used shRNA and the Knipe group used a pool of four siRNAs. Both knockdown methods targeted all PML isoforms at the common N-terminus but by different sequences. It has been reported that different PML isoforms are known to be recognized and degraded by ICP0 via totally distinct biochemical mechanisms [83,95,96]. Gu and colleagues showed that in HSV-1 infected cells, a single SIM located at ICP0 residues 362-364 was essential for the degradation of PML isoforms II, IV, and VI. However, for the degradation of PML I, this SIM was found dispensable. Instead, PML I-interaction domains located within ICP0 residues 1–83 and 245–474 redundantly facilitate the interaction between ICP0 and PML and the degradation of PML I [95,96]. The obvious differential recognition of PML isoforms by ICP0 suggests that PML isoforms may play distinctive roles in HSV-1 infection. With that in mind, a plausible explanation that can reconcile these contradictory results from the Everett and Knipe reports is that PML isoforms may be targeted with different efficiency when the sequences and amounts of siRNA are different. This may further alter the balance in the protein networking of PML within ND10 and thereby lead to the opposite results.

Another study implying a promotive role of ND10 in HSV-1 replication comes from the Roizman group, in which a CRISPR/Cas knockout of the PML gene caused a significant reduction in the replication of the wild-type HSV-1 at low MOI [22,97]. Roizman and colleagues further characterized these PML-/- cells and found that Sp100 was less induced upon IFN treatment compared to the parental cells. ICP0 was also less produced and did not properly colocalize with the Sp100/Daxx aggregates, which resulted in an ineffective Sp100 degradation in the PML-/- cells [22]. These results have revealed an effector role of PML that mediates the IFN response in HSV-1 infection but also indicated the dependence of HSV-1 on PML for a more effective replication. PML isoform II has been shown to specifically interact with the transcription factors NFκB, IRF3, and STAT1 to promote the expression of IFNβ and ISGs [98], suggesting that different PML isoforms can mediate specific reactions in cellular response triggered by HSV-1 infection. Whether these reactions are intertwined with other regulatory pathways via the PML associated proteins in ND10 will need further investigation.

**Figure 1:**
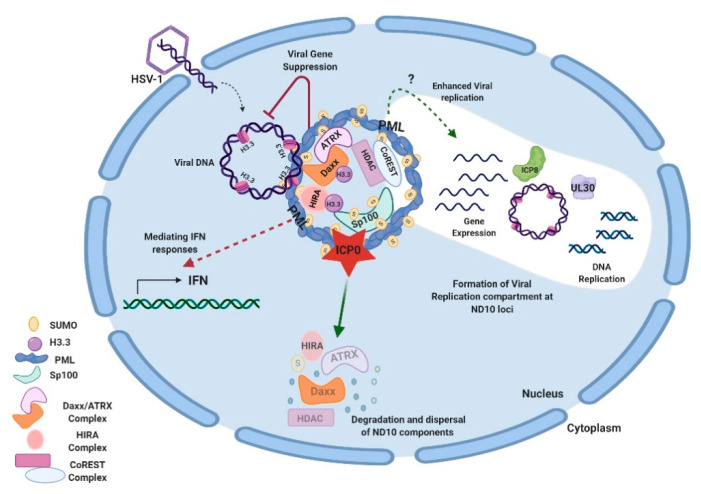
Interactions between HSV-1 and ND10 in the lytic cycle. HSV-1 injects its naked double-stranded DNA into the nucleus of the infected cell. ND10 components converge to the incoming DNA and impose suppression on the viral DNA. Specifically, Daxx/ATRX, and HIRA complexes deposit H3.3 histones on viral DNA to create the heterochromatin silencing. PML is an effector to mediate the IFN response in HSV-1 infection. Newly expressed ICP0 counteracts gene repression by degrading PML and Sp100 and dispersing ND10. Viral genes get expressed and replication compartments form at the ND10 loci after the ND10 dispersal. Viral proteins such as ICP8 (ssDNA binding protein) and UL30 (viral DNA polymerase) are typical markers for the replication compartments. The efficiency of viral replication can be reduced or enhanced by a PML depletion, depending on experimental conditions. Components of the CoREST complex are recruited to ND10 and reside in the viral replication compartments. This figure was created with BioRender.com.

Studies on additional ND10 components have shown that ND10 functions in HSV-1 infection are associated with the chromatin remodeling of viral DNA. HSV-1 ejects naked DNA into the nucleus. The incoming DNA immediately encounters histone deposition to form a chromatin-like viral genome [99,100,101]. Cabral et al. showed that ATRX was required in the maintenance of viral chromatin stability but not involved in the initial viral chromatin formation [102]. In another study, HIRA (histone cell cycle regulator), a histone H3.3 chaperone, was found to be recruited to ND10 and bind to the HSV-1 genome to mediate the H3.3 deposition early in HSV-1 infection [103,104]. HIRA localization to ND10 is cell-type dependent [103,104]. Therefore, it is not yet clear whether the HIRA-dependent histone deposition and the ATRX-mediated chromatin maintenance are sequential events or whether they have any cooperativity in the early infection. A chromatin corepressor, CoREST, and its associated proteins are also recruited to ND10 in early HSV-1 infection [105]. In all of the above cases, chromatin formation and repressor association of the HSV-1 genome, which bring inhibitions to the viral gene expression, are regarded as parts of the host’s intrinsic antiviral defenses. The degradation of PML and the disruption of ND10 by ICP0 is considered a key viral countermeasure to release the chromatin repression imposed by ND10. Additional viral counteractions to release the ND10 restrictions include viral microRNAs that downregulate the ATRX expression [106]. An interesting phenomenon associated with the triangular relation of HSV DNA, ND10, and viral counteractions is that HSV-1 replication compartments evolve at the ND10 foci after the disruption of ND10 [79,107,108]. The observation that CoREST and CoREST-associated proteins are also found in the replication compartments [109] raises further questions for the roles of proteins recruited to ND10 during the HSV-1 infection. Is it possible that some of these proteins are recruited to ND10 in order to mediate some of the pro-viral functions of ND10 in the establishment of HSV-1 transcription/replication? An overview of ND10-HSV-1 interactions in the lytic cycle is depicted in Figure 1.

### 4.2. HSV Latent Infection

Besides the lytic infection phase, HSV-1 and HSV-2 establish lifelong latent infection in ganglia neurons, which is the etiological cause of recurrent infections in HSV pathogenesis. Latent HSV infection can be experimentally reproduced in animals [110]. However, due to the limited availability of ganglia neurons and the infection heterogeneity in these neurons, ND10 functions in HSV latently infected animals are largely unclear. Much of the latency studies are performed with HSV-1 and remain descriptive. Fluorescent staining showed that in a latently infected ganglion, about half of the infected neurons contained one single spot of HSV-1 DNA enwrapped by the donut-shaped ND10 body, where the latency-associated transcript (LAT) was undetectable. The other half of the infected neurons in the same ganglion contained multiple spots of HSV-1 DNA, where at least one of the spots was colocalized with ND10, and the LAT expression spread out to overlap with all spots [111]. Although viral mRNA profiles in these heterogeneously infected neurons have not yet been reported, it is quite reasonable to postulate that viral gene expression will be different around the HSV genome within the distinct spot patterns. Whether and how ND10 nuclear bodies in these spot patterns play differential roles in HSV latency maintenance or latency reactivation remain to be seen.

HSV IE-deficient viruses can form quiescent infection in cultured fibroblasts, in which the viral genome persists in cells for a long period of time but the viral genes are tightly silenced. The quiescent genome does not respond to the general reactivation stimuli but can be reactivated by the introduction of IE proteins such as ICP0 or by the treatments that perturb heterochromatin. Because of the experimental simplicity and reproducibility in the quiescent infection it is considered an alternative model to the animal latency model and a good tool to mimic the molecular relationship between the latent HSV genome and ND10 [112]. With this system, Cohen et al. showed that the quiescent viral genome of HSV-1 was associated with two histone H3.3 chaperone complexes, Daxx/ATRX and HIRA, in multiple loci. They redundantly chromatinized all classes of viral genes including the LAT locus with the histone H3.3 modified by the repressive K9 trimethylation mark [113]. The heterochromatinization of LAT in this study is in accordance with the previous observation that a latent genome associated with an ND10 body in a single spot pattern silences the LAT expression in vivo [111]. However, how this quiescence is related to the heterogeneous spot pattern in mice remains unanswered. PML was shown to play an important role in the silencing of the quiescent genome. In its absence, the colocalization of Daxx/ATRX with the quiescent viral genome was reduced, and the H3.3 load on the multiple loci was significantly decreased [113]. The disruption of ND10 integrity in cells harboring the quiescent viral DNA by the ectopic expression of ICP0 or by the treatment of histone deacetylase (HDAC) inhibitors led to the reactivation of the quiescent genome [113,114]. These results confirmed the repressive function of ND10 in the quiescent infection. With the obvious lack of neuronal supportive cells and surrounding tissues, the quiescent infection in monolayer fibroblasts cannot completely recapitulate the HSV-1 latency. However, the results are still indicative although do not necessarily reflect the full picture. The newly developed technology of 3D organoids derived from iPSC (induced pluripotent stem cells) [115] may become a better model for the mechanistic studies of HSV-1 latency, and a comprehensive role of ND10 in the latent HSV-1 infection remains to be seen.

### 4.3. VZV

Varicella-zoster virus (VZV) is the third human α-herpesvirus that is restricted by ND10 [116]. Results showed that PML and Daxx knockdown increased virus production [116], whereas overexpression of PML isoform IV inhibited it [117]. ORF61, the VZV ortholog of ICP0, was capable of disrupting ND10 via the SIMs located in its N- and C-termini [118]. ORF61 of VZV does have a RING finger domain [119], but it does not mediate PML degradation [116]. This means HSV-1 and VZV use different mechanisms to overcome the ND10 restrictions. Interestingly, PML IV inhibited viral yield by forming a cage-like structure around the newly formed viral capsids, likely to prevent the nuclear egress process [117], reflecting a role for ND10 in virus assembly and maturation. The relationship between VZV and the other PML isoforms is not clear, whereas beneficial roles of ND10 have not been reported in VZV infection.

## 5. Dual Role of ND10 in β-Herpesvirus Infection

### 5.1. HCMV

Human cytomegalovirus (HCMV) is a member of the Betaherpesvirinae subfamily, which replicates in a variety of differentiated cells including the epithelial and endothelial cells and establishes its latency in the less differentiated hematopoietic cells [120,121]. Similar to the traditional antiviral functions found with HSV-1, ND10 components Daxx and Sp100 are known to recruit ATRX and HDACs to the major immediate early promoter (MIEP) and bring the repressive histone marks to the HCMV viral chromatin [122,123,124,125,126]. Knockdown of PML, Daxx, or Sp100 improves the expression of HCMV IE genes and enhances viral replication at low MOI [124,126,127,128,129], whereas PML is also found to mediate IFN response and promote ISG expression [130].

In a more complex manner than that of HSV-1, two viral proteins, an immediate-early protein IE1 and a tegument protein pp71, have been found to counteract the ND10 restrictions in HCMV infection [131,132,133]. IE1 is found to colocalize with ND10 and interact with PML [132,133,134]. However, PML is not degraded during HCMV infection, but the ND10 bodies are dispersed in an IE1-dependent manner [132,133,134,135]. Schilling et al. showed that ectopic IE1 expression alone prevented de novo PML SUMOylation [136], suggesting that the lack of PML SUMOylation may be the cause of ND10 disruption. A recent report also identified IE1 as an E3 ligase that can induce the degradation of Sp100 A [137], suggesting multiple means of IE1 to counteract the antiviral functions of ND10 in HCMV infection. With the functional homology between ICP0 and IE1, IE1 can partially compensate for the loss of ICP0 in HSV-1 infection [138]. In addition to IE1, the tegument protein pp71 also counteracts the ND10 restrictions by binding to Daxx to induce Daxx degradation and dislodging ATRX [125,126,131,139,140,141].

IE1 has also been reported to interact with PML and therefore disrupt the formation of STAT/HDAC complex on the ISG promoters, as another pathway of the viral counteractions associated with the ND10 restrictions on HCMV replication [130]. However, a recent systematic mutational scanning of IE1 conducted by Paulus et al. showed that clustered charge mutants in the two regions previously defined for PML-interaction [130,142] led to the downregulations of IE1 [60], which triggered a reevaluation of IE1 functions in the PML-related IFN response. With the systematic mutations, Paulus et al. identified a new IE1 mutant, cc172–176, which lost the PML binding activity but had a protein level comparable to that of the wild-type IE1. Results from IE1cc172–176 showed that the loss of PML interaction disabled IE1 from dispersing PML from the ND10 bodies but it did not substantially affect the HCMV replication, even at low MOI [60]. More interestingly, while IE1cc172–176 did not bind to PML it can still bind to STAT. Therefore, infection by a recombinant HCMV carrying the IE1cc172–176 mutant induced less IFN expression compared to that of the wild-type virus [60], suggesting that the IE1-PML interaction to disperse PML from ND10 may be associated with the enhancement of ISG expression, instead of being a viral counteraction to dampen the IFN response. Whether there is a trade-off for HCMV to disperse PML, for example, to gain the interaction to certain ND10 factors, remains to be seen. It would also be interesting to investigate whether the individual PML isoforms are differentially dispersed in HCMV infection and to examine their consequences.

As in α-herpesviruses, ND10 functions in the latent HCMV infection are under-investigated. PML is known to be required for the maintenance of HCMV latency. LUNA (the latency-associated protein) is a deSUMOylase that removes the SUMO marks from PML, which triggers the ND10 disruption and promotes the HCMV reactivation. Mutations in the deSUMOylase domain of LUNA prevent the HCMV reactivation, whereas PML deletion can rescue the reactivation of a recombinant HCMV containing the LUNA mutant [143], suggesting that ND10 bodies have a role in keeping the balance between the maintenance and the reactivation of HCMV latency.

### 5.2. HHV-6A, HHV-6B, HHV-7

Human herpesviruses 6A, 6B, and 7 (HHV-6A, HHV-6B, HHV-7) are the other human β-herpesviruses that can infect several types of cells but are mainly characterized for their growth in T lymphocytes [121]. With the short history of T cell culture and T lymphotropic virus research, the relation between HHV-6/7 and ND10 is poorly understood. Viral gene suppression by ND10 has been reported with HHV-6A, but viral counteractions are only speculated based on the observation that the number of ND10 foci is reduced during the infection [144]. In HHV-6B infection, IE1B is found to colocalize with ND10 through the SUMO–SIM interaction, which further implies the viral modifications of ND10 in infection [145].

While many herpesviruses keep their genomes as episomes during latency, HHV-6A/B integrate their latent genome into the host telomeres to maintain the viral copy number during the cell division [146]. Recently, Collin et al. reported that in PML knockout cells the percentage of HHV-6B genome integration was significantly lowered [147], indicating the contribution of PML to the genome integration of HHV-6B. This result is in line with the fact that a subset of PML colocalizes with telomeres and participates in the maintenance of telomere stability [148], suggesting that HHV-6B may take advantage of the PML function in telomeres to facilitate the viral genome integration for the long-term persistence.

## 6. Dual Role of ND10 in γ-Herpesvirus Infection

### 6.1. EBV

Epstein–Barr virus (EBV) is a member of the Gammaherpesvirinae subfamily. It establishes lytic infection in epithelial cells and B lymphocytes, but the predominant mode of infection is the latency in B lymphocytes. The EBV latent infection is a common cause for B cell malignancy, whereas abortive lytic infections are also associated with the EBV-related oncogenesis [149,150]. Tsai et al. showed that in primary B lymphocytes, EBV DNA was localized next to the ND10 components, Daxx and ATRX [151], which may reflect the host attempt to silence the incoming genome. A viral tegument protein, BNRF1, was found to interact with Daxx, which led to the disruption of the Daxx/ATRX complex from localizing to the viral genome. Consequently, the loading of H3.3 histones across the viral genome was reduced, and the active chromatin mark H3K4me3 in the tested regions was enhanced [151,152], leading to the expression of EBNA2, a characteristic of the pre-latency phase [151]. Interestingly, BNRF1 can enhance the replication of ICP0-null HSV-1 or pp71-deficient HCMV, confirming that BNRF1 can compensate for the loss of ICP0 and pp71 in counteracting the antiviral effects of ND10 [153]. Another pre-latency marker, EBNA-LP, which is the specific coactivator of EBNA2, was also found to interact with Sp100 and displace it from ND10, suggesting that EBNA-LP further modulates the ND10 composition in the establishment of latent EBV infection [154].

Unlike the entrapment of the HSV-1 genome within ND10 during HSV-1 latency, early investigation of the latently infected cells derived from Burkitt’s lymphoma showed that EBV episomes had no association to the intact ND10 bodies stained by Sp100 [155]. Moreover, in the latently infected nasopharyngeal cells, EBNA1, the latency-associated protein necessary for the stable persistence of EBV episomes [156], was found to trigger the proteasomal degradation of PML [157]. These results clearly indicate that the EBV DNA replication alongside cell division is ND10-independent. In the meantime, ND10 involvement in the EBV-associated malignancy may be more relevant to the ND10 functions in DNA damage response or apoptosis, which are known to be regulated by the EBV proteins such as EBNA1 [158].

Latent EBV cell lines derived from the EBV-associated tumors can be induced for reactivation by HDAC inhibitors such as sodium butyrate or cell activator tetradecanoyl phorbol acetate (TPA) [159]. In the transition from latent to lytic infection, ND10 nuclear bodies were found to be disrupted, and ND10 components were sequentially dispersed, with Daxx and Sp100 leaving ND10 in the early stages of the lytic cycle but PML remaining within ND10 till the late stages [61,155]. Mere disruption of ND10 by arsenic treatment [160] or by the depletion of Daxx/ATRX [152] caused the EBV reactivation and lytic gene expression, indicating that ND10 bodies have suppressive functions in the EBV lytic infection. Two lytic gene transactivators, BZLF1 and BRLF1, may counteract the ND10 restrictions during the lytic induction, inasmuch as transfection of BZLF1 alone in the latently infected cells can induce EBV to reactivate [159,161], and transfected BRLF1 can bind to SUMO to induce the disappearance of ND10 [162]. Whether these proteins manipulate ND10 in vivo, however, needs further investigations.

Although these results suggest that EBV is restricted by ND10 in the lytic phase, other reports have implied beneficial roles of ND10 in EBV replication as well. Wang et al. showed that PML interacted with the minor viral capsid protein BORF1, which further recruited other capsid proteins to the ND10 bodies to facilitate the viral assembly. Depletion of PML significantly reduced the virion production without a change in the expression level of the capsid proteins [163]. Additional studies indicating positive roles of ND10 in EBV replication came from the fact that SM, an early nuclear protein of EBV involved in mRNA processing, physically interacted with Sp110 B to increase the stability of viral transcripts [164,165,166].

### 6.2. KSHV

Kaposi’s sarcoma-associated herpesvirus (KSHV), also known as human herpesvirus 8 (HHV-8), is the other member of the human γ-herpesviruses. This virus establishes the latent infection in endothelial cells and B lymphocytes, leading to the transformation of several types of cancers [121,167]. A GFP-expressing recombinant KSHV showed that knockdown of PML or Sp100 gave a higher number of GFP positive cells in the primary infection, indicating the restrictive effects of ND10 components on the incoming KSHV [168]. To further understand the role of ND10 in the de novo KSHV infection, Grundhoff and colleagues examined the influences of KSHV infection on the morphology and composition of ND10. They found that similar to what was shown with EBV, the latent KSHV genome did not colocalize with ND10, whereas the depletion of PML, Sp100, or Daxx did not affect the latency establishment [169]. Further analyses, however, showed that during latency, although the number and size of Sp100-positive ND10 nuclear bodies were moderately increased, the low-salt extractable Sp100 protein disappeared [169]. Based on the facts that Sp100 knockdown accelerates the heterochromatin formation of the KSHV genome while not affecting the latency establishment and the latency-associated nuclear antigen (LANA) has the ability to enhance the SUMOylation of Sp100 [169], a likely model is that LANA functions to sequester soluble free Sp100 into the insoluble ND10 condensates through SUMOylation and therefore enhances the KSHV heterochromatin formation during the latency establishment and maintenance.

Similarly to EBV, KSHV latently infected cells can be induced to reactivate in vitro. Recently, beneficial roles of PML in induced KSHV lytic infection have also been reported. Studies in the KSHV harboring cells derived from primary effusion lymphoma (PEL) showed that PML knockout decreased the KHSV DNA replication and virion production in the reactivation, while PML overexpression enhanced the viral replication [170]. It is not yet clear whether the positive role of PML on KSHV is in the virus assembly like that of EBV.

Several interactions between KSHV proteins and the ND10 components have suggested the inhibitory potential of ND10 in the KSHV lytic phase. For example, KSHV tegument protein ORF75, a homolog to BNRF1 of EBV, colocalizes with PML and disperses Daxx and Sp100 from the ND10 foci [168]. KHSV early protein K8 is a SUMO E3 ligase that colocalizes with PML and catalyzes the SUMOylation of p53 [171,172,173], which may be relevant to the senescence regulation in the process of KSHV lytic replication [174]. Rta, a viral transcription factor essential for the lytic gene expression that colocalizes to ND10, exhibits SUMO-targeting E3 ubiquitin ligase activity and degrades PML in vitro [62]. However, how these KSHV proteins coordinate their interactions with ND10 in the context of KSHV infection remains unclear.

## 7. Conclusions

**Figure 2:**
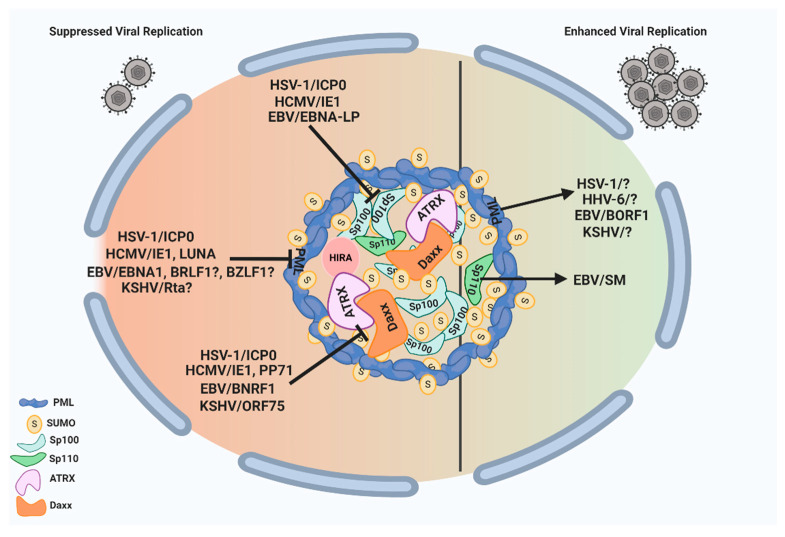
ND10 nuclear bodies serve as multifunctional nuclear hubs with both pro- and anti-viral outcomes. On the one hand, ND10 nuclear bodies mediate intrinsic and innate immune responses to herpesvirus infection through some of their residents such as PML, Daxx/ATRX, and Sp100. All subfamilies of human herpesviruses express proteins that counteract these suppressive functions of ND10. These viral proteins are listed on the left side of the figure, and their specific targets are indicated by the block signs. On the other hand, herpesviruses have evolved to benefit from some of the ND10 proteins as well. The proteins that can improve herpesvirus replication are indicated by the arrows pointing to their specific target viruses on the right side of the figure. The unknown viral proteins involved in the pro-viral interactions or viral proteins with a possible role in antagonizing ND10 are indicated by question marks in the figure. This figure was created with BioRender.com.

ND10 nuclear bodies can serve as multifunctional nuclear hubs to orchestrate a wide array of cellular functions. They are armed with multiple suppressive factors such as PML, Sp100, Daxx/ATRX, and HIRA complex that can limit the growth of herpesviruses through (i) chromatin repression of the incoming naked DNA and (ii) mediation of the host IFN response. In turn, herpesviruses have evolved to counter the ND10 restrictions by encoding viral proteins, and sometimes microRNAs, that can disturb the ND10 integrity via the degradation or redistribution of the ND10 components. Since many of the herpesvirus proteins counteracting ND10 are essential for viral replication at low MOI, they are promising targets for antiviral drug development. Although the molecular mechanisms are not yet clear, recent results have shown that herpesviruses are also capable of exploiting ND10 nuclear bodies to enhance the lytic growth or long-term maintenance in the host. These actions include the requirement of PML in EBV capsid assembly and in the viral productivity of HSV-1 and KSHV, the importance of Sp110 B in the EBV mRNA accumulation, and the involvement of ND10 in the maintenance of the latent HHV-6B genome. The pro-viral functions of ND10 have also been reported in many non-herpes viruses including adenoviruses and papillomaviruses [175,176,177,178,179,180,181], suggesting the possible common features of ND10 effects in DNA virus infections. HSV-1, with rather established culture systems and a clear protein functional profile, is an ideal model virus to lead the investigation into virus–ND10 interactions. A total delineation of the complex involvement of ND10 in HSV-1 infection may bring a fundamental breakthrough in the understanding of molecular relations between viruses and their hosts. An overview of the interplay between herpesvirus proteins and ND10 components with pro- and anti-viral functions is illustrated in Figure 2.
